# *Bertiella studeri* Infection, China

**DOI:** 10.3201/eid1201.050579

**Published:** 2006-01

**Authors:** Xin Sun, Qiang Fang, Xing-Zhi Chen, Shou-Feng Hu, Hui Xia, Xue-Mei Wang

**Affiliations:** * Bengbu Medical College, Bengbu, China;; † Anhui Provincial Key Laboratory of Infection and Immunity, Bengbu, China

**Keywords:** Bertiella studeri, Human infection, China, letter

**To the Editor:** *Bertiella* is a genus of tapeworm in the family *Anoplocephalidae*, many species of which exist as parasites of nonhuman primates. Two species of the genus, *Bertiella studeri* and *B. mucronata*, can infect humans (1). More than 50 cases of human infection have been recorded, and the geographic distribution of cases shows that the tapeworm exists in countries in Asia, Africa, and the Americas. We report a *B. studeri* infection in a person; to our knowledge, this case of bertiellosis is the first in China.

The patient was a 3.5-year-old Chinese boy from Suzhou City, Anhui Province. The boy had a 6-month history of frequent abdominal pain. His parents had noticed living "parasites" in his feces for 3 months; a segment of the worm was expelled every 2 or 3 days. According to the symptoms, doctors at the local hospital diagnosed his condition as *Taenia solium* infection and prescribed praziquantel, but no drug was available in the hospital or local drugstores. Consequently, the parents brought the child to Bengbu Medical College for further diagnosis and treatment.

The patient appeared healthy; routine medical examination showed normal heart, lung, liver, and spleen, and he had no fever. Though the patient had intermittent epigastric pain, the abdomen was soft and tender. A total of 133 proglottids were collected from the feces. Their average length was 0.1 cm, and the total length of all proglottids was 13 cm; each segment was 0.68–1.10 cm in width. Eggs (N = 53) were examined microscopically; they were roundish or oval, an average of 45.31 μm diameter (range 37.93–50.00 μm), and clearly showed typical pyriform apparatus, with visible hooklets (Figure). Other laboratory examinations showed hemoglobin level of 110 g/L, erythrocytes 3.9 × 10^12^ cells/L, and leukocytes 8.0 × 10^9^ cells/L. Although 2 species can parasitize humans, the geographic distribution and egg size of these species differ (2). *B. mucronata* has smaller eggs and is found only in the New World. On the basis of the size of the proglottids (3), larger eggs with pyriform apparatus and hooklets, and geographic distribution, the infecting cestode was identified as *B. studeri*.

**Figure:**
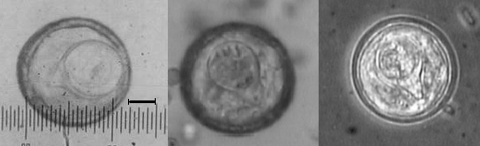
Eggs collected from proglottids. Left panel shows the length of the egg, scale bar = 10 μm; middle panel shows the hooklets in the egg; right panel shows the pyriform apparatus in the egg (under convert microscope).

The origin of infection was not confirmed; the only clue was that the boy's parents had once raised tame monkeys in a zoo. When the boy was 2 years old, he often played in the wildland, which is part of the zoo near the forest, and frequently fed and played with the captured monkeys. Further questioning showed that the boy had also been in frequent contact with wild monkeys. We could not confirm whether he had been infected by eating monkey food contaminated with mites.

The lifecycle of the cestode requires 2 hosts; nonhuman primates are generally the final host, while oribatid mites are the intermediate host, in which the infective cysticercoid of the cestode develops. Orbatid mites may exist in soil to maintain natural infection, and the definitive host is infected by eating or otherwise coming into contact with contaminated soil or food. Animal infection has been recorded in some provinces in China, and human bertiellosis has been recorded in Sri Lanka (4), Saudi Arabia (5), Vietnam (6), Japan (7), India (8), Thailand, Malaysia, and other Asian countries. However, according to the most recent Chinese authoritative text, Human Parasitology (9), no human bertiellosis has been recorded in China. Humans are infected by unconsciously swallowing infected mites, and in Mauritius, children were infected by eating guavas that had fallen on the soil (10). Other human infections may have occurred, but infected persons may have had mild symptoms and not noticed expelling the segments, so local doctors may have considered the cases to have been caused by a common cestode. To prevent human bertiellosis, the relationship between human cases and the natural host must be investigated.
